# Residual Pain in the Context of Selecting and Switching Biologic Therapy in Inflammatory Rheumatic Diseases

**DOI:** 10.3389/fmed.2021.712645

**Published:** 2021-08-17

**Authors:** Florian Berghea, Camelia Elena Berghea, Dumitru Zaharia, Andreea Iulia Trandafir, Elena Cristina Nita, Violeta Maria Vlad

**Affiliations:** ^1^Department of Rheumatology, Carol Davila University of Medicine and Pharmacy, Bucharest, Romania; ^2^Sf. Maria Hospital, Bucharest, Romania; ^3^Marie Curie Emergency Children's Hospital, Bucharest, Romania

**Keywords:** residual pain, biologic therapy, rheumatod arthritis, ankilosing spondylitis, remission, switch, post remission syndrome, disease modifying anti-rhuematic drugs

## Abstract

For many years, inflammatory rheumatic diseases (IRDs) represented a source of disappointment in medical care caused by the mediocre efficacy of the available treatments. Some of these diseases, like Rheumatoid Arthritis (RA) or Ankylosing Spondylitis (AS), caused fear in the general population, especially due to associated joint deformities and subsequent disabilities. However, in the last 20 years, a new successful class of antirheumatic drugs has become available: biologic Disease-Modifying Antirheumatic Drugs (bDMARDs). Due to this innovative treatment, the days are over when joint and spine deformities defined the condition of a person with RA or AS. Nonetheless, expectations are higher today, and other clinical problems, (not entirely solved by bDMARDs), seem to drive the drug selection during the span of rheumatic diseases. Most of these issues are covered by the term “unmet needs.” One of the most intriguing of such needs is the residual pain (RP) in patients that are otherwise in the biological remission of the disease. Present in a significant proportion of the patients that enter remission status, RP is poorly understood and managed. In recent years, new data has become available in this area and new conceptual clarifications have occurred. In this review, we explain the various nature of RP and the necessity of treatment diversification in such situations. All in all, we believe this condition is far more complex than simple pain and includes other clinical aspects, too (like fatigue or mood changes) so the terms Post-Remission Syndrome (PRS), and PRS pain might be more appropriate.

## Introduction

IRDs represent a heterogeneous group of musculoskeletal disorders associated with localized or systemic inflammation resulting in characteristic connective tissue and internal organ damage. In one of the largest prevalence studies performed in the USA, the prevalence of all forms of arthritis was 21.6% in the general population with smaller proportions for inflammatory arthritides: RA (0.5–1%), AS (0.5%), overall Spondylarthritides—SpA (0.3–1.3%) or Systemic lupus erythematosus—SLE (0.07–0.14%) (1). The management of IRDs consists of various forms of inflammation suppression; the approach has been refined in recent years according to the “treat to target” concept—with clinical and biological remission as the final aim. Therapeutic options include corticoids and non-steroidal anti-inflammatory drugs, biologic/ conventional synthetic/ targeted synthetic DMARDs (b/cs/tsDMARDs). Both clinical efficacies and general safety profiles of bDMARDs placed them at the top of rheumatologists' preference. However, a certain amount of joint destruction, fatigue, suboptimal physical or mental functions, disability, inability to work or RP are still present in the rheumatic patient's life and demand new or adapted managements solutions (2, 3). These are all included under a generic definition of unmet needs in managing rheumatic patients. Although, the therapy is better now than 20–30 years ago, with improved outcomes, some of these unmet needs tend to become more important now than in the past. Different kinds of pains, and especially RP that a patient in biological remission is still experiencing, are some of the most important unmet needs for these patients and—obviously—solid drivers of drug selection.

## Pain—Present Conceptual Clarifications

It is well-known that pain is the clinical symptom that receives the greatest attention, not only in rheumatic diseases but in the whole medical spectrum. Recently the notion of pain underwent major conceptual clarifications. The International Association for the Study of Pain (ISAP) revised its definition of pain in 2020 (4) to differentiate between pain from nociception: “An unpleasant sensory and emotional experience associated with, or resembling that associated with, actual or potential tissue damage”; this definition implies that pain does not always need the presence of nociception stimulation.

**Figure 1:**
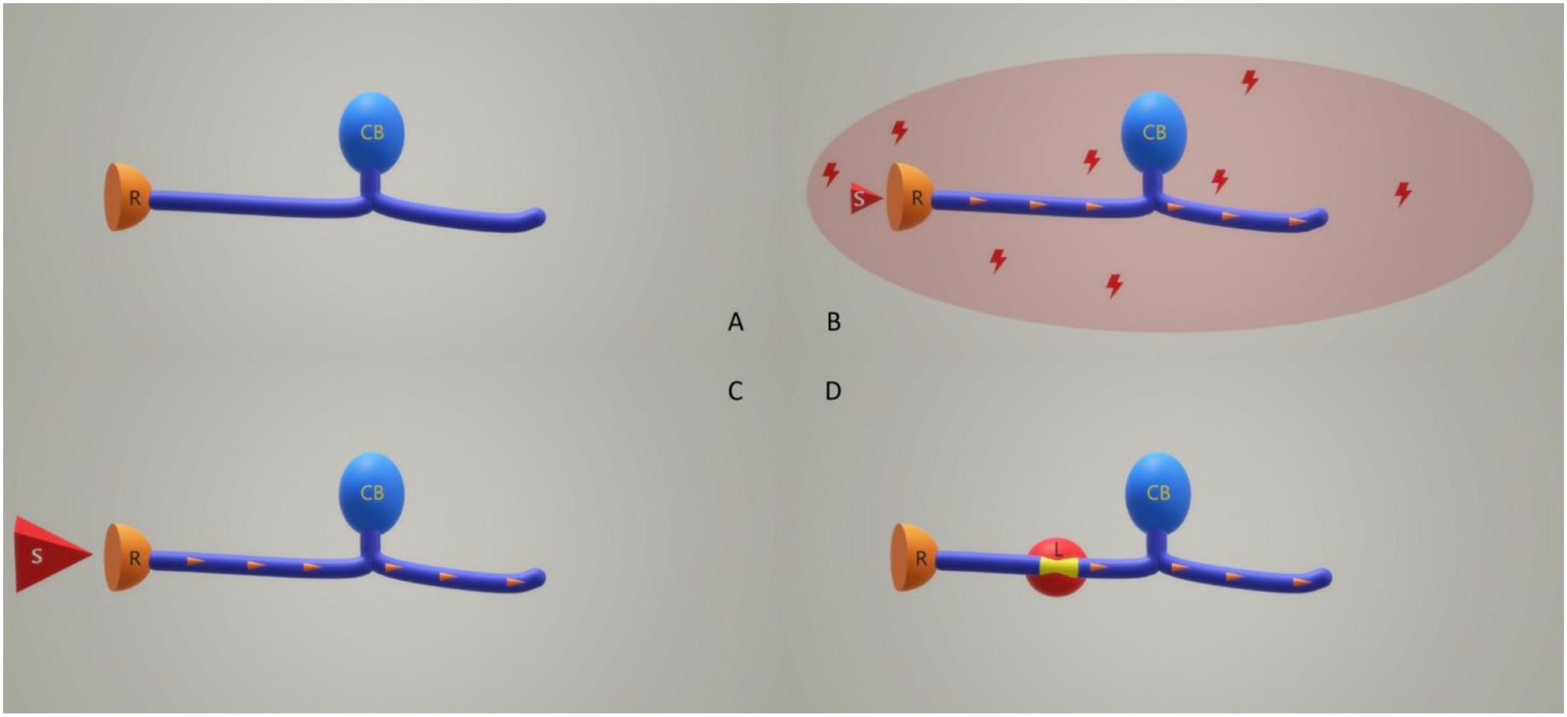
Relation between pain type and sensory neurons functionality. **(A)** Normal sensory neuron without any stimulus. **(B)** Nociplastic pain situation: a normal sensory neuron surrounded by a hostile microenvironment is receiving a subliminal stimulus. **(C)** Nociceptive pain situation: a normal sensory neuron receives a proper magnitude stimulation. **(D)** Neuropathic pain situation: an injured sensory neuron does not receive any stimulation of the receptors, or the stimuli are insignificant. In **(B–D)** situations the result is similar: an electrical impulse (orange arrowheads) is created. R, receptor; CB, cellular body; S, stimulus; L, the lesioned area of the sensory neuron.

Apart from nociceptive pain (generated by proper stimulation of nociceptive sensors in terms of duration and intensity), the present classification includes two other different entities: neuropathic pain (pain caused by a lesion or a disease of the somatosensory nervous system) and nociplastic pain (“pain that arises from altered nociception despite no clear evidence of actual or threatened tissue damage causing the activation of peripheral nociceptors or evidence for disease or lesion of the somatosensory system causing the pain” (5, 6) – Figure 1).

The relative new entity, nociplastic pain, was included to differentiate the nociceptive pain that arises in a normal somatosensory system following a proper stimulation from the one that appears following a subliminal stimulation of healthy neurons that live in a hostile microenvironment (toxic, inflammatory, etc.,). In many cases neuropathic and nociplastic pains coexist; proper profiling of pain in the rheumatic patient is recognized as paramount for focused successful management (7, 8). The differentiation between acute and chronic pain was kept in the last ISAP revision. Furthermore, chronic pain syndromes, defined as persistent or recurrent pain lasting ≥3 months (9), have been separated into primary and secondary domains. In primary syndromes, pain is seen as a disease while in secondary syndromes pain is viewed just as a symptom—a part of a different disease (10). The present ISAP classification included chronic musculoskeletal pain (CMP) in the group of chronic pain syndromes; it is defined as chronic pain arising from musculoskeletal structures such as bones or joints; it is divided into primary and secondary pain. Primary CMP is a pain that cannot be explained by a known disease or a damaging process (e.g., low back pain) (11). Secondary CMP has a clear causative etiology: (a) a local or systemic inflammatory disease (in relation with infection, crystal deposition, autoimmune or autoinflammatory processes), (b) local structural musculoskeletal changes, and (c) diseases of the nervous system which may cause musculoskeletal problems (e.g., neurologic disorders that generate prolonged muscular contractions). As a consequence, all pain related to inflammatory rheumatic diseases (no matter the level of clinical or biological activity of these diseases) is considered secondary CMP.

## How Do We Assess RP?

To affirm the existence of RP in a rheumatic patient the rheumatologist has to perform a concomitant assessment of pain and disease activity. Performing pain assessment is still not possible with an objective imagistic method (such as MRI or ultrasound) (12). However, even simple pain measurement instruments such as the visual analog scale (VAS), numeric rating scale (NRS), verbal rating scale (VRS) together with several subscales like Arthritis Impact Measurement Scales (AIMS/AIMS2) and the bodily pain subscale of the Medical Outcome Study Short-Form Survey 36 bring sufficient information about the magnitude of pain (13–15). In the particular case of neuropathic pain, the PainDETECT questionnaire seems to be increasingly preferred (16, 17).

Regarding IRDs activity assessment we have instruments with universal acceptability. In RA, disease activity score 28 (DAS28), composite scores like Simple Disease Activity Index (SDAI), Clinical Disease Activity Index (CDAI), Patient Activity Scale-II (PAS-II), and of course, subjective measures like visual analog scale (VAS) are accepted. All these scores correlate well with joint damage and physical function (18, 19). In AS, the most used instruments are the Bath AS Disease Activity Index (BASDAI), the AS Disease Activity Score (ASDAS), Bath AS Functional Index (BASFI), Bath AS Metrology Index (BASMI). Although, RP is reported in various papers between 11 and 40 mm measured on 100 mm VAS (20), RP is defined as pain with amplitude >20 mm in a patient that entered the remission phase of his IRD (21).

## The Etiology of RP

Pain and inflammation are in a close link (22) but inflammation means more than pain; it means fatigue, fever, anxiety, joint stiffness etc., Once the inflammation diminishes, the whole disease picture changes accordingly, making it difficult for the patients to focus on and describe subtle changes in level or pattern of pain. It becomes reasonable then to wonder if the RP is just the same initial pain, now with a different extension and intensity, or if it is a completely newly developed entity. It is perfectly acceptable to question the inflammatory origin of RP when biological markers and clinical symptoms of inflammation, are gone. Some other hypotheses have been generated covering both nociplastic and neuropathic mechanisms of pain and giving less extent to inflammatory pain. In this regard fibromyalgia and its potential participation in RP have recently gained importance. Data suggests that fibromyalgia in conjunction with inflammatory rheumatic diseases has a prevalence between 15 and 30%, larger than in the general population −2% (23). Could fibromyalgia explain the RP? A large US-based study (23) investigated the development of fibromyalgia in patients with RA: 9739 subjects have been followed up for a total of 42 591 patient-years. The incidence rate of fibromyalgia was 5.3 per 100 patient-years, with no differences between sexes. The fibromyalgia criterion was met in 7% of men and 8.1% of women at the end of the study. Along with the study, using the accepted diagnostic criteria for fibromyalgia, many patients passed the border of diagnostic (several times) being in or out of the diagnostic category. This observation raises the question of the real nature and long-term stability of RP (or, at least, of the fibromyalgia part of it). But fibromyalgia (a nociplastic type of pain) cannot be solely responsible for explaining RP in RA patients. There are also reports that raise questions about different pain mechanisms (e.g., neuropathic) indicated in RA patients. Using self-reported questionnaires like painDETECT, PROMIS 29, Widespread Pain Index/Symptom Severity Scale on 169 RA patients; a recent study concluded that neuropathic and fibromyalgic pain are different (24).

## RP in Rheumatic Patients and the Concept of Post-Remission Syndrome

Joint pain is a universal finding in patients with RA but can persist even in people who enter inflammatory remission status (25). Concerning the large discrepancies between objective measures of inflammation and reported pain shown in patients with RA, increasing evidence supports a role for aberrant pain processing, including peripheral and central pain sensitization in the pathogenesis of pain in RA (26). In addition, proofs for (at least partial) neuropathic characteristics of pain in RA, in the absence of local inflammation or of local destruction of tissues, are now available. The amplification of nociceptive information transmission may explain the negative association between chronic pain and DAS28 in RA patients with low disease activity (27). Recent data suggested a strong presence of neuropathic pain in rheumatic conditions. In a Spanish-based rheumatic outpatient study, neuropathic pain was identified in 12% of subjects (28). In a Swedish population-based case-control study investigating the long-term correlation between pain and objective inflammation after RA diagnosis, the correlation was lost at the three-month follow-up, suggesting that the uncoupling of pain and inflammation may develop early after diagnosis and treatment initiation in RA patients (29). Other data suggests that a sub-group of RA patients could develop non-inflammatory pain in their early disease and consequently not respond to anti-inflammatory therapy. These patients are characterized by a higher baseline disability, higher tender joint count in 28 joints and higher DAS28 combined with less baseline inflammation according to erythrocyte sedimentation rate (30). Thus, it is reasonable to believe that right from the beginning of the disease, some patients will develop fibromyalgia pain and others a non-fibromyalgia, non-inflammatory pain. In fact, despite the use of multiple analgesic medications, about 50–60% of patients with RA in remission according to DAS28 have pain on VAS>10 that emphasizes the need to take a different, non-anti-inflammatory drug approach (31).

Looking toward the other major inflammatory rheumatic disease, AS, the situation is similar. In a large UK population-based study of pain in adults, chronic widespread pain (CWP) was more commonly found in the AS subgroups than in RA or the general population (32, 33). In AS, neuropathic pain seems to be highly prevalent in patients with longer disease courses, when compared to nociceptive pain seen in those with recent disease. AS patients with neuropathic pain components usually show significantly higher disease activity and pain severity, more depression, more structural damages in the spine, wider peripheral manifestations including enthesitis and peripheral arthritis, and lower quality of life than those without a neuropathic pain component (34).

Last but not least, evidence suggests a different phenotype for those who experience RP, somehow independent from peripheral stimulation: central pain prone phenotype (12). This sub-group is more likely to develop chronic pain and a better response to centrally-acting drugs (e.g., tricyclics, serotonin-norepinephrine reuptake inhibitors, gabapentinoids) than to current anti-inflammatories.

**Figure 2:**
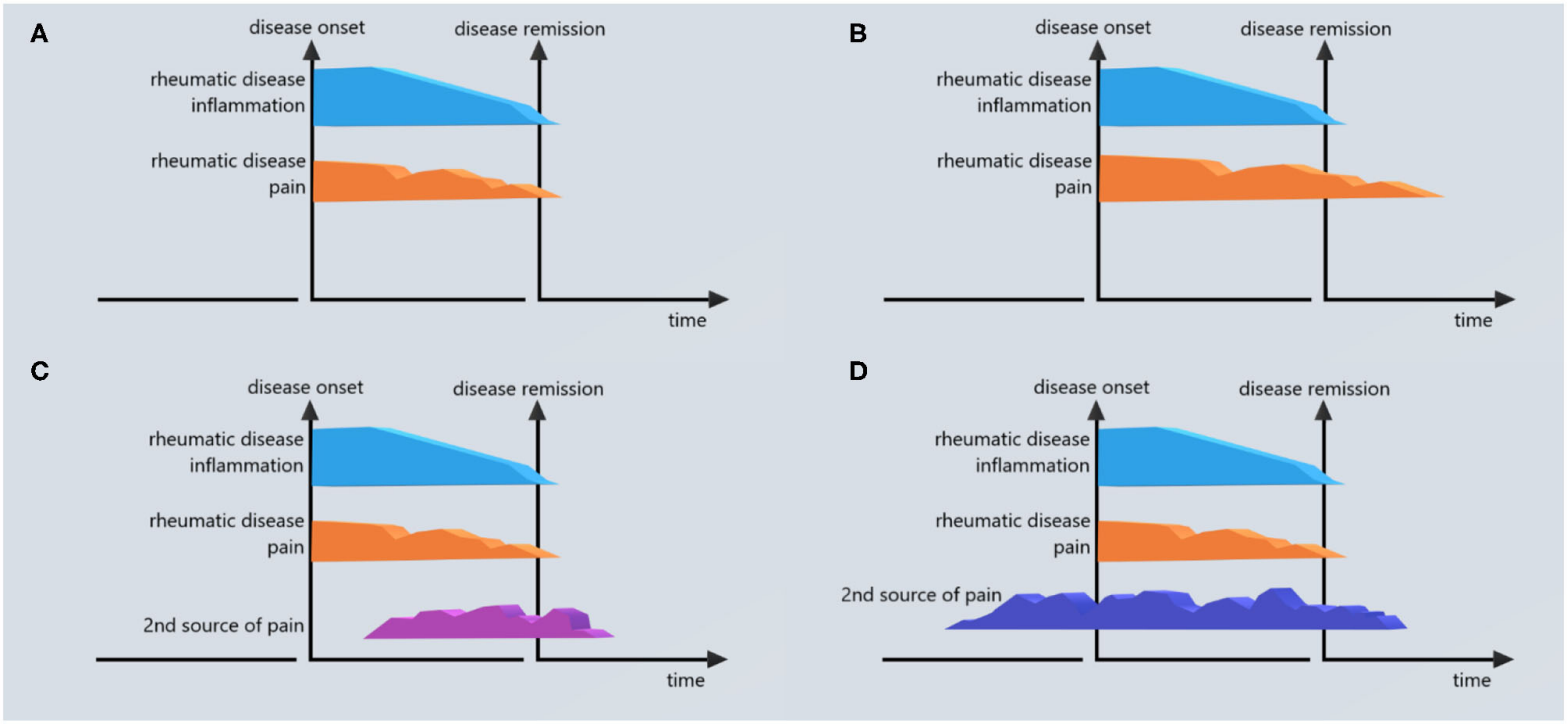
Different scenarios of RP in IRDs. **(A)** No RP (pain after remission <20 mm VAS). **(B)** RP is the unresolved part of the pain generated by inflammatory rheumatic disease (IRD). **(C)** RP comes from a second source developed during the evolution of the rheumatic disease and independent of the pain generated by IRD. **(D)** RP comes from a second source with the onset that preceded the onset of IRD; this source does not end after the remission of IRD.

Concluding, if a patient in remission is still experiencing pain, several scenarios are possible: (a) – the disease is *not* in remission and RP represents the remains of what was once the inflammatory onset pain; (b) – the disease *is* in remission but the patient developed concomitantly a second kind of pain, superposed during the evolution of the IRD; (c) – the RP represents a secondary effect of IRD at the level of pain system structures (in a similar way the AA amyloidosis is a secondary development in some rheumatic patients) – Figure 2. We do not know now what is the most probable scenario so the terms “RP” and “remaining pain” might be misleading by referring exclusively to the first scenario and excluding the non-nociceptive or the development of a second pain from the context. At the same time, RP is not a singular symptom in patients that enter the remission status but a part of a larger clinical picture that includes fatigue, mood changes, functional disability, sleep disorders (35–38). That's why a more appropriate term might be “post-remission syndrome—PRS.” Such a term addresses the wider spectrum of possible causes and informs both the patient and the rheumatologist about the necessity of a continuation of sustained medical care during the “remission” phase of the disease.

## The Role of RP in the Selection and Discontinuation of Biological Therapy

Qualitative studies in RA patients identified several patient concerns about the efficacy of treatment: pain, physical function, social role, morning stiffness, and well-being (39). Only some of the patients accept RP; many others demand a change in their therapy. Due to its ability to influence the way participants adapt to and cope with the experience of pain response, acceptance became a significant process for the applied clinical context. Pain-related anxiety has proven to be the best predictor of pain acceptance (40). Moreover, a British study that included almost 15,000 RA patients revealed a strong connection between discontinuation of TNF alfa-inhibitors at 1 year (defined as complete stop or just the switch to another TNF alfa-inhibitor) and baseline pain (41). The authors hesitated to indicate pain as the main driver of biologic therapy discontinuation but emphasized that it predicted discontinuation of drugs due to inefficacy—not due to adverse events.

An expression of the incapacity of anti-inflammatory therapy to effectively manage rheumatic pain can be seen in the proportion of rheumatic patients that continue to use opioids despite the introduction of bDMARDs. A US study revealed that 40% of RA patients use opioids in various forms and 12% use them chronically (42). Another study that followed 2,330 RA patients between 2007 and 2015 showed similar data (38.8% used opioids constantly throughout the whole study period); the introduction of TNF alfa-inhibitors diminished the percentage of opioid users from 54 to 51% (some patients ceased opioids after initiation of TNF alfa-inhibitors therapy, but a similar proportion started opioids on the same occasion).

## Are all DMARDs Equal in the Front of RP?

Although, the precise profile of RP is not entirely decrypted, the need for a solution encouraged comparisons between bDMARDs, csDMARDs, and tsDMARDs - JAK inhibitors in terms of the amplitude of RP and persistence during therapy. Head-to-head studies are rare but existing reports tend to support the idea that not all drugs are equally efficient in the prevention of RP. Taylor et al. in 2019 (43) analyzed RP in relation to Baricitinib, Adalimumab, and Methotrexate treatments in RA patients and concluded that Baricitinib was superior to Adalimumab and Methotrexate. Fautrel et al. in 2020 (44) analyzed the pain reducing ability of Baricitinib, Tocilizumab, Adalimumab, Tofacitinib, and Methotrexate and concluded that Baricitinib is superior to the comparators. Bykerk et al. in 2019 (45) compared Sarilumab and Adalimumab and concluded that no difference exists in terms of RP but Sarilumab is superior in reducing the unacceptable pain. A clear explanation of such potential differences between various DMARDS in pain modulation and especially in the reduction of RP is not available yet. However, we have some starting evidence. We know that TNF-α can directly sensitize nociceptive fibers (46). In addition, TNF-α and IL-6 seem to work *via* Janus kinase/signal transducer and activator of transcription (JAK-STAT) pathway (47); other studies have shown that IL-6 has a powerful pro-nociceptive effect (48).

## Discussions

RP has recently become a clinical entity of increasing interest. Far from being elucidated yet, the present data support a mixed origin of RP including chronic nociceptive, neuropathic, and nociplastic pain. A poor response to DMARDs and other anti-inflammatory drugs seems to indicate a non-inflammatory origin. Fibromyalgia (a disease that is far from being elucidated, too) may explain RP in a group of subjects but not in all. Having in mind the coexistence of other clinical modifications in this context, we support the idea that RP is just a part of a larger entity that can be defined after the remission of the disease is declared: Post-Remission Syndrome – PRS. The wider spectrum of residual symptoms in low disease activity (LDA) patients was firstly reviewed by Ishida et al. in 2016 when 68 studies have been analyzed – pain was mentioned in 25 of them, being the most frequently reported residual symptom (20). The PRS possible composition is a strong indication for widening our therapeutic arsenal beyond the anti-inflammatory drugs. However, going back to the mechanism of action of bDMARDs, csDMARDs, and tsDMARDs, it might be useful to understand the real capacity of each molecule to prevent the development of PRS or at least of the PRS pain. For the time being, it seems as though, this feature differs from molecule to molecule. In a not-so-far future, it will be possible to have this issue in mind when a bDMARDs is selected.

Apart from a self-evident research interest in the clarification of the nature of PRS pain, further research questions demand better answers. Is PRS pain a common outcome of all patients receiving an improper or incomplete treatment scheme, or is it just a matter of individual differences? The question is open. Is it reasonable to switch from one bDMARDs to another just because of the PRS pain? Most probably not (at least if other non-anti-inflammatory solutions have not been used). Could we rank DMARDs based on their ability to prevent PRS? Certainly, there is a strong interest and we will see this sooner than we think. What are the phenotypic differences and what is the role of central sensitization in PRS pain? The existing research will bring new data. Are our disease activity evaluation instruments able to reflect the whole spectrum of inflammatory and non-inflammatory pain we meet in IRDs? We don't know, and it is wise to perform a detailed pain profiling of each subject by using different instruments and to adjust the therapeutic scheme accordingly. Although, many rheumatologists are reluctant to consider themselves to be pain specialists and go beyond the anti-inflammatory therapy (49) in the management of PRS pain, they could derive inspiration from other specialties (intensive care, neurology, psychiatry) and try *their* solutions. Tricyclic antidepressants, serotonin, and norepinephrine reuptake inhibitors, or selective serotonin reuptake inhibitors are just a few classes of drugs efficient in pain therapy rarely used by rheumatologists. A combination of bDMARDs, csDMARDs or, tsDMARDs and some of these drugs might have a superior effect on PRS pain than a simple switch of various bDMARDs.

## Author Contributions

All authors listed have made a substantial, direct and intellectual contribution to the work, and approved it for publication.

## Conflict of Interest

The authors declare that the research was conducted in the absence of any commercial or financial relationships that could be construed as a potential conflict of interest.

## Publisher's Note

All claims expressed in this article are solely those of the authors and do not necessarily represent those of their affiliated organizations, or those of the publisher, the editors and the reviewers. Any product that may be evaluated in this article, or claim that may be made by its manufacturer, is not guaranteed or endorsed by the publisher.
